# Toward graphene textiles in wearable eye tracking systems for human–machine interaction

**DOI:** 10.3762/bjnano.12.14

**Published:** 2021-02-11

**Authors:** Ata Jedari Golparvar, Murat Kaya Yapici

**Affiliations:** 1 Faculty of Engineering and Natural Sciences, Sabanci University, TR-34956 Istanbul, Turkey; 2 Sabanci University SUNUM Nanotechnology Research Center, TR-34956 Istanbul, Turkey; 3 Department of Electrical Engineering, University of Washington, Seattle, WA 98195, USA

**Keywords:** electrooculography (EOG), flexible electronics, graphene, human–computer interaction (HCI), human–machine interface (HMI), personal assistive device (PAD), wearable smart textile

## Abstract

The study of eye movements and the measurement of the resulting biopotential, referred to as electrooculography (EOG), may find increasing use in applications within the domain of activity recognition, context awareness, mobile human–computer and human–machine interaction (HCI/HMI), and personal medical devices; provided that, seamless sensing of eye activity and processing thereof is achieved by a truly wearable, low-cost, and accessible technology. The present study demonstrates an alternative to the bulky and expensive camera-based eye tracking systems and reports the development of a graphene textile-based personal assistive device for the first time. This self-contained wearable prototype comprises a headband with soft graphene textile electrodes that overcome the limitations of conventional “wet” electrodes, along with miniaturized, portable readout electronics with real-time signal processing capability that can stream data to a remote device over Bluetooth. The potential of graphene textiles in wearable eye tracking and eye-operated remote object interaction is demonstrated by controlling a mouse cursor on screen for typing with a virtual keyboard and enabling navigation of a four-wheeled robot in a maze, all utilizing five different eye motions initiated with a single channel EOG acquisition. Typing speeds of up to six characters per minute without prediction algorithms and guidance of the robot in a maze with four 180° turns were successfully achieved with perfect pattern detection accuracies of 100% and 98%, respectively.

## Introduction

Eye tracking technologies have many applications ranging from behavioral analytics to healthcare. For a brand leader, the prospect of seeing the world literally through consumer’s eyes, as opposed to relying on traditional market research methods, is the reason that makes eye tracking a clear winner in understanding the triggering subconscious facts in decision making. In entertainment and virtual reality applications, eye tracking enables a whole new interaction method with contents, and it could also add new security measures through retinal scanning.

In healthcare, eye tracking enables devices to help ease a disabled individual’s challenging life using their eye motions. Thousands of people suffer from extreme disabilities such as severe cerebral palsy and amyotrophic lateral sclerosis (ALS), which deprive them of muscular abilities. It is estimated that approximately 450,000 people worldwide are diagnosed with ALS, and in the United States, the incidence of ALS is two per hundred thousand people [1]. Therefore, the advancement of eye tracking technologies is critical for developing assistive devices and human–computer interaction (HCI) platforms for people with disabilities.

Current technologies for eye tracking, however, by being either invasive, expensive, or bulky [2–4] do not address the requirements of a readily usable product in HCI intended for widespread use; and fail to meet the fundamental characteristics of wearability in terms of size miniaturization, low-power operation, ergonomics, and aesthetics. For example, although camera-based eye tracking setups solve the invasivity issue and display long-term functionality, they are hardly affordable due to their hardware (e.g., camera) and image processing requirements. Additionally, in videooculography, the camera has to be positioned at a location suitable to capture eye movements (EMs), which limits the portability of such systems. Therefore, effort has been placed to investigate different methods to utilize EMs in wearable applications.

To this end, electrooculography (EOG) is an economical (a typical EOG setup could be assembled at a cost of under 100 EUR [5]), non-invasive, and reliable method for acquiring biopotential signals around the eyes, and may overcome the limitations of prior technologies. EOG is essentially based on a simple model of the human eye as a dipole with a permanent potential difference between its forward and backward facing spots (i.e., the cornea-retinal potential of 0.4–1.0 mV where the cornea is positive) [6]. This potential difference sets up an electrical field in the tissues surrounding the eye, which generates an electric field [7]. Two electrodes around the eyes can locate the field vector rotation, which creates a more positive charge in the electrode that the cornea is approaching [8]. The biopotentials resulting from eye activity, which are in fact waveforms that relate the dipole fluctuations to the type of eye movement, are referred to as electrooculograms; while the specific technique for recording is known as electrooculography [9]. Electrooculograms also occur in total darkness, when the eyes are closed, even in visually impaired people [10].

So far, several EOG-based rehabilitation systems were developed to mitigate everyday life challenges of and provide some means of communication for people with locked-in syndromes who have extremely limited peripheral mobility but still retain their eye motor coordination [10]. Similarly, basic deliberate eye movements such as saccades (i.e., fast eye movements), fixations (i.e., the duration between two saccades when the gaze is fixated at a point), and blinks have been used for hands-free operation in HCI and human–machine interfaces (HMIs) [11]. With the help of HCI and HMIs one can emulate a computer mouse [12], type using a virtual keyboard [13], drive a wheelchair or control a robotic arm [14], change TV channels [15], and even improve user experience on virtual reality gaming [16] or smartphone operation [17]. Along these lines, in the healthcare domain, as part of a hospital alarm system, EOG-based switches provided immobile patients with a safe and reliable way of signaling an alarm [18]. Other promising studies reported the direct input of numbers, letters of the English alphabet, and Japanese Katakana symbols by eye movements to further help users to communicate complicated messages in a relatively short time [19].

However, despite the various demonstrators of wearable EOG devices in the literature, which prove that EOG is a measurement technique that is reliable, easy to operate, and can be made cosmetically appealing, EOG-based devices still struggle to penetrate the wearables market, and their full potential has not been realized due to limitations of the sensing electrodes [20–22]. The combination of graphene with ordinary textiles presents a viable solution to this limitation [23]. Therefore, this work reports an alternative eye tracking system based on monitoring the ocular biopotential. It demonstrates the first graphene textile-based wearable personal assistive device for interacting with machines and controlling objects. With the developed wearable system prototype, seamless “eye-operated” remote control of objects is achieved, including cursor motion on screen for typing of text and the motion of a four-wheeled car; both of which demonstrate the vast potential of graphene textiles as an enabler of wearable assistive technologies.

## Materials and Methods

### Synthesis of graphene textiles and electrode preparation

**Figure 1:**
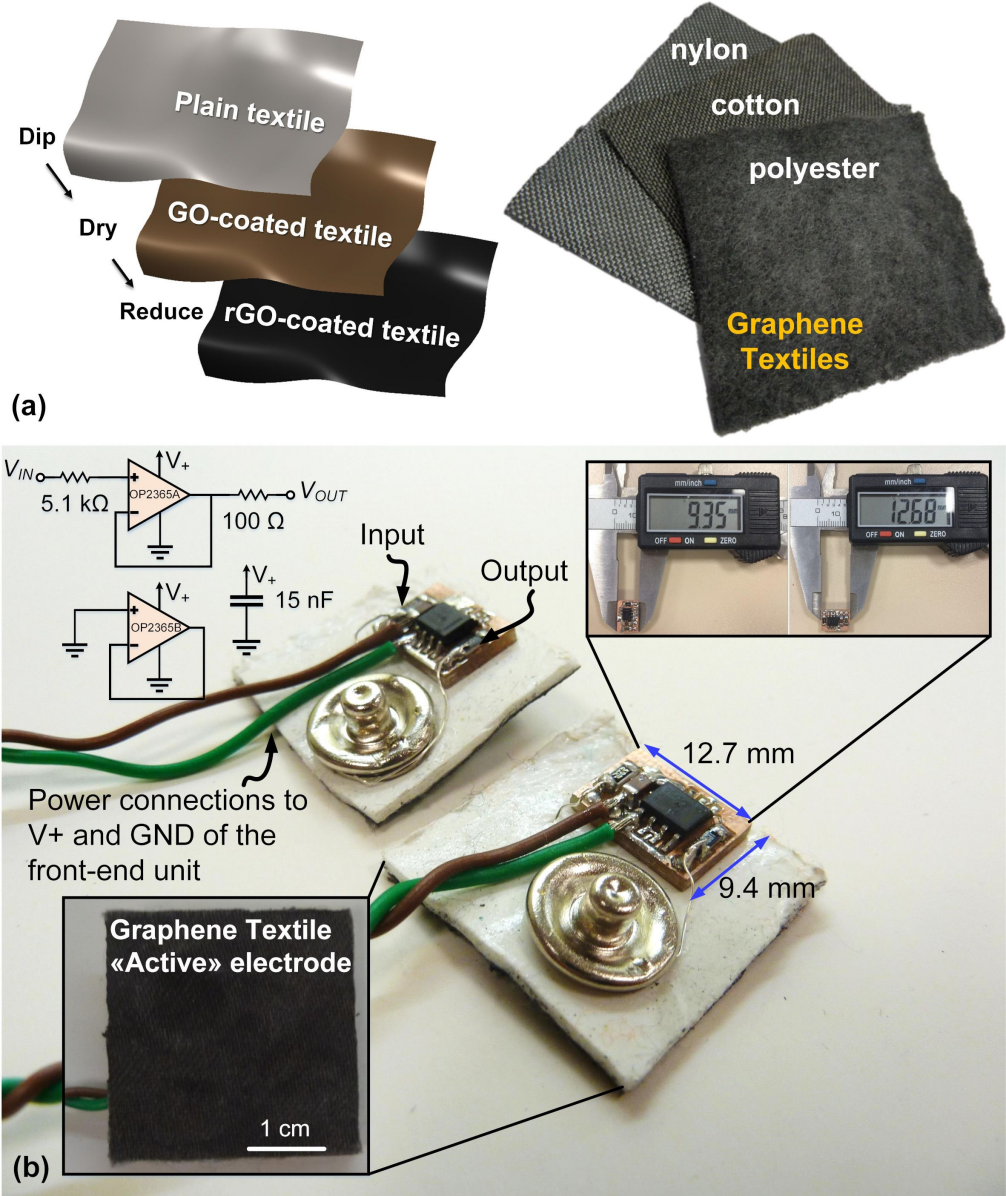
(a) Schematic of the three-step process to obtain conformal graphene coatings on a variety of textiles including nylon, cotton, and polyester; (b) image of the graphene textile “active” electrode with integrated buffer circuitry along with component values and circuit schematic. *V*_in_ is connected to the graphene textile, and *V*_out_ is connected to the snap button. Both are used to provide the connection of the “active” textile electrode to the signal acquisition unit; insets show the front side of the electrode assembly with the graphene textile and the dimensions of the miniaturized-PCB on the rear side of the electrode.

The developed process to synthesize conductive graphene textiles is based on a low-cost and scalable, three-step approach in which conformal layers of graphene were formed on various fabrics including nylon, polyester, cotton, and Kevlar (Figure 1a). The textiles were first dipped into graphene oxide (GO) suspension prepared by the modified Hummer’s method, dried to allow for the layering of GO on the textiles, treated by reducing agents (e.g., hydrazine or hydrogen iodide), and rinsed with distilled water to form a stable graphene coating on the textiles. The prepared graphene textiles were then cut into pieces (ca. 3 × 3 cm) and mounted on flexible, polyethylene-based foam paddings, which were sandwiched between metallic snap fasteners in order to establish an electrical connection with the front-end circuitry. In this way, “passive” graphene textile electrodes were formed, which can be directly used to capture surface biopotentials without further modification. Conductivity measurements showed resistance values of the textiles between 1 and 10 kΩ and skin-electrode impedance values from 87.5 kΩ (at 10 Hz) to 11.6 kΩ (at 1 kHz). Additionally, since the operation of the textile electrodes relies on charge flow, moisture and sweat can increase the interface conductivity of the skin electrodes and provide an even better signal-to-noise ratio (SNR) in long-term monitoring applications in contrast to “wet” electrodes, the functionality of which degrades over time [23].

However, one common issue in dry electrodes is the relatively high skin-electrode contact impedance, which causes susceptibility to physical movements and power line interferences resulting in signal distortions. While the flexible, foldable nature of textile electrodes promotes wearability, it can adversely lead to dynamic contact conditions and, thereby, motion artifacts in the acquired signal. A strategy to minimize this effect, which was investigated in this work, is to reduce the impedance of the signal source by utilizing a buffer amplifier, which essentially converts the high-impedance signal to a low-impedance signal. Figure 1b shows the circuit schematic and the components for building “active” graphene textile electrodes. The components are an operational amplifier (OPA2365, Texas Instruments, USA) with a high input impedance, two resistors, and one capacitor. A small-sized printed circuit board (ca. 0.9 × 1.3 cm) was designed and implemented to demonstrate a proof of concept. It allowed for robust integration and an electrical interface between the buffer-circuit components and the graphene textiles to form “active” electrodes.

### Wearable system prototype

**Figure 2:**
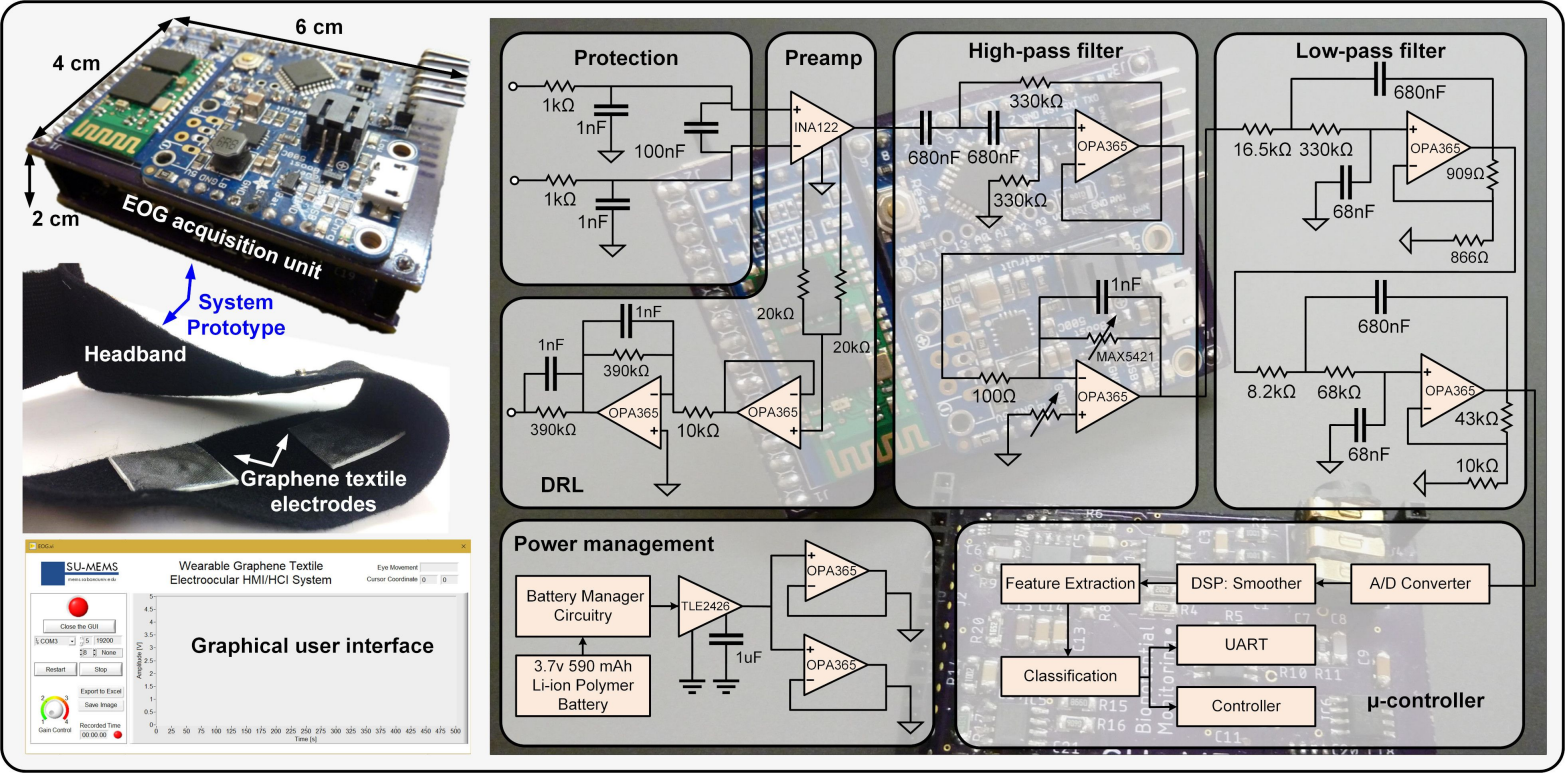
Overview of the system prototype showing the detailed hardware-level schematic of the portable, battery-powered, EOG acquisition unit including on-board filtering and gain stages, power management section, microcontroller unit to process and stream data wirelessly to a computer along with a custom-designed user interface, and the wearable headband with soft graphene textile electrodes.

One of the fundamental obstacles regarding the actual wearability of electronic systems is the lack of robust integration schemes for the interface between soft sensing electrodes and rigid electronic components. We have addressed this issue by following a system-level design perspective. A miniaturized, battery-powered, portable EOG acquisition unit with wireless data transmission capability was realized and directly integrated into a wearable headband, which also contained the graphene textile electrodes (Figure 2). The electrodes were mounted in a newly introduced manner such that potential crosstalk between channels was eliminated. Also, electrode count and hardware complexity were minimized by removing the vertical acquisition channel without loss of control commands triggered by vertical EMs [24–25]. The readout electronics could be implemented in a small form factor (ca. 4 × 6 cm) that is easily attachable on a wearable custom-made headband. The front half of the headband was kept fairly non-stretchable to maintain the electrode locations on the forehead; while the rear side was made of elastic straps enabling size adjustment to ensure a stable skin–electrode contact.

As shown in the detailed hardware schematic in Figure 2, the circuit has second-order and fourth-order Butterworth high-pass and low-pass filters with cut-off frequencies of 0.5 Hz and 10 Hz, respectively, both based on Sallen–Key topology. The preferred instrumentation amplifier (INA122, Texas Instruments, USA) is specifically designed for battery-powered applications with the capability of running with a single supply. Similarly, the other operation amplifiers used in the design were selected due to their single-supply and rail-to-rail features (OPA2365, Texas Instruments, USA) and are suitable for portable applications. For adjusting the gain in the post-amplification stage, a digitally programmable voltage divider (MAX5421, Maxim, USA) was used so that the gain can be configured at the software level through a graphical user interface (GUI). As a common practice in portable devices nowadays, a lithium-ion/polymer battery with a rating of 3.7 V and 500 mAh was chosen to power the system. The battery charge management circuitry and DC–DC boost converter were based on a MCP73831 (Microchip, USA) and a TPS61090 (Texas Instruments, USA), respectively.

To split the regulated 5 V, a rail splitter (TLE2426, Texas Instruments, USA) was used, which is essentially a voltage divider with a buffer circuitry to prevent it from becoming unbalanced. The currents that can be typically handled by power splitters are small (20–40 mA). Hence, additional buffers with higher current ratings were accommodated in the power management unit as a safety margin, despite the typically low levels of currents drawn by the signal acquisition unit. To adjust the settings and to stream data to a computer, a popular off-the-shelf Bluetooth module (HC06) was used. A custom-designed GUI based on LabVIEW (National Instruments, USA) was implemented at the receiver end, enabling user-friendly operation, calibration, gain adjustment, and event monitoring.

In the current version of the embedded system, the Bluetooth coverage is ca. 5 m. The power consumption of the circuitry is ca. 80 mW when the Bluetooth device is not paired and ca. 150 mW when it is paired, which allows the battery to run in the paired mode for about 4 h continuously without recharge. These measurements can be further improved by implementing sleep-mode functions to a microcontroller and upgrading the Bluetooth module to a Bluetooth low-energy (BLE) module, such as HM-10, which has low power consumption and offers a higher range up to several tens of meters.

## Results and Discussion

Different eye movements along with a detection and classification algorithm having a success rate ranging from 85% up to 100% for detecting eleven different patterns over an hour-long EOG recording experiment [25] were utilized to develop a graphene textile-based wearable eye mouse and a wearable assistive system to control movements of a four-wheeled robot. The wearable eye mouse controlled a cursor on a PC screen and sent *x*–*y* coordinates of the cursor to the GUI. On the computer, the GUI uses the “SetCursorPos” and “mouse_event” functions of the Microsoft Windows User32.dll library to control cursor movement and blink actions, respectively. Swift horizontal eye movements (to the left or right) control the horizontal movement of the cursor in two directions. In contrast, slow eye movements control the cursor motion in vertical directions. Finally, voluntary blinking mimics the mouse click action. When swift or slow eye movements occur, the cursor starts to move in the defined direction at a preconfigured speed until it reaches the edge of the display or until a different eye movement changes the cursor motion. For instance, the cursor will move toward the left side after a swift left eye movement and stop and execute a click action when a blink of the eye occurs. Some additional actions were implemented for better control, such as “cursor stop”, which stops the cursor motion at any point without causing a click action. For instance, when the cursor moves, either a swift left or a swift right eye movement can stop the cursor movement. In this way, the user can achieve accurate position control and fine-tune the cursor location when needed. Likewise, the “cursor snap” option was defined to allow for a rapid, discretized transition of the cursor from its existing location to the nearest key in the specified direction of motion.

**Figure 3:**
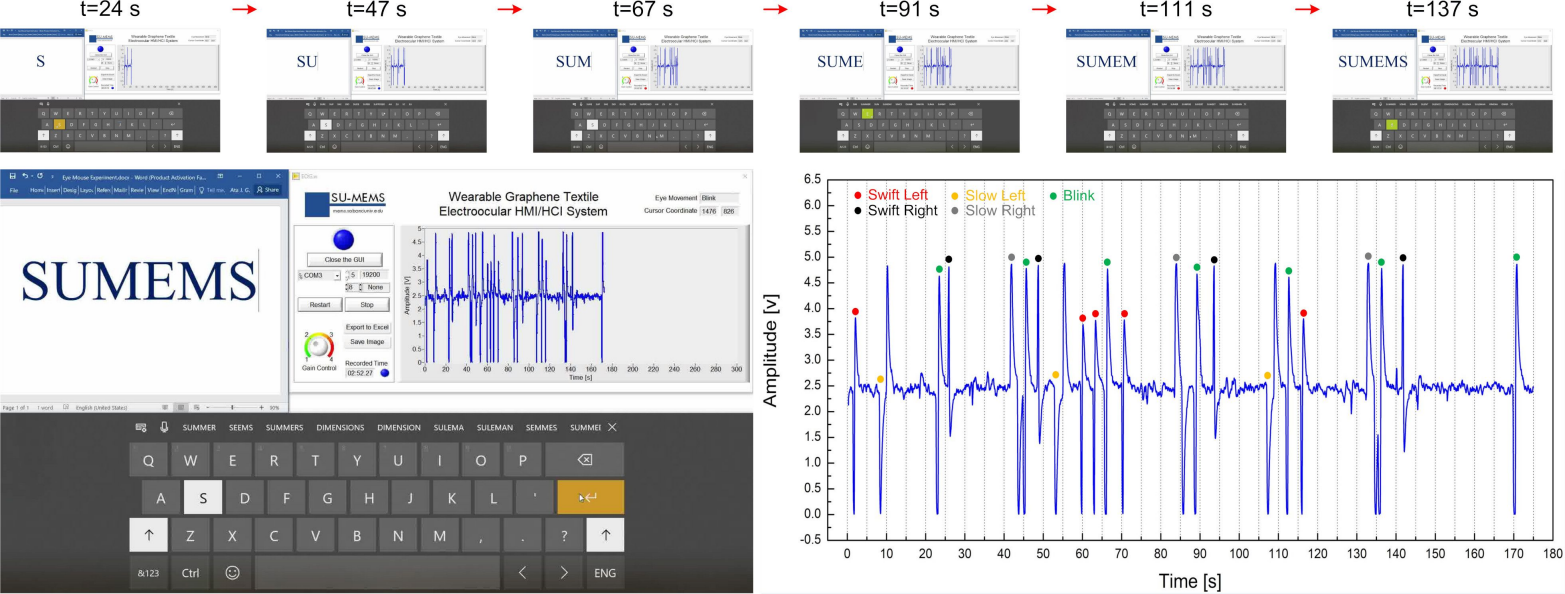
The first testing scenario shows the plot of induced EOG signals with inserted interpretations for each eye movement, which are used to mimic the movements of a mouse cursor to write “SUMEMS” (Sabanci University MEMS Group).

To evaluate the performance of the graphene textile-based wearable eye mouse, two different tests were carried out. In the first case, a single participant was trained and asked to type a specific word, while the lateral or transverse movements of the cursor on the screen were continuous unless terminated by another command. In the second case, “cursor snap” was activated and five participants were requested to type five randomly selected character sequences of equal length. Figure 3 demonstrates the first testing scenario with the developed eye mouse and the recorded electrooculograms together with their interpretation. The aim here is first to open Microsoft Word Office and the Microsoft Windows virtual keyboard, to write a word, and, finally, to stop the GUI solely by eye movements. The different eye movements and corresponding cursor actions specific to the eye movements are summarized in Table 1, along with a representative sequence of movements needed to type “SUMEMS” and the measured duration for each step.

**Table 1:** Summary of eye movement types with corresponding cursor actions and a representative typing sequence.

Sequence	Type	Action	Duration [s]

1	swift left	left direction	6.5
2	slow left	down direction	14.7
3	blink	click “S”	instantaneous
4	swift right	right direction	15.7
5	slow right	up direction	3.3
6	blink	click “U”	instantaneous
7	swift right	right direction	4.9
8	slow left	down direction	6.1
9	swift left	stop	3.5
10	swift left	left direction	3.0
11	blink	click “M”	instantaneous
12	swift left	left direction	13.7
13	slow right	up direction	5.8
14	blink	click “E”	instantaneous
15	swift right	right direction	13.3
16	slow left	down direction	5.1
17	blink	click “M”	instantaneous
18	swift left	left direction	17.0
19	slow right	up direction	3.4
20	blink	click “S”	instantaneous
21	swift right	right direction	28.9
22	blink	click “Enter”	instantaneous

Speed and initial starting point of the cursor, and several other options are configurable in the GUI settings. In the first trial, the cursor speed (pointer speed on screen) was kept slow and set to 1 pixel every 30 ms, which, on average, is translated to one character every ca. 23 s. This resulted in a total typing time of over 2 min for a 6-character word.

The cursor speed was gradually increased in subsequent trials to identify the upper limit on typing speed with the developed wearable eye mouse system. The fundamental constraint on increasing the cursor speed is the inherent transient response of electrooculograms and the detection algorithm to account for this behavior. On the occasion of an eye movement, a certain amount of time is required for the corresponding EOG signal to complete its waveform and reach a steady state, upon which the detection algorithm classifies the performed eye movement and executes the designated action. For instance, considering the case of a blink, which is attributed to the mouse click or “select” action, at cursor speeds above a maximum threshold, there is the possibility that the user attempts to click and select a specific letter but misses it due to the latency in command execution. In our trials with a trained participant, the cursor speed was optimized such that the participant was able to correctly type one character per approx. 10 s, which corresponds to a typing speed of six characters per minute (CPM), a more than two-fold increase in the typing speed compared to the first trial. This is a reasonably good speed for a thresholding-based algorithm approach in which a pattern detection accuracy of up to 100% was achieved, and in alignment with earlier EOG-based spellers, which range from 2.4 to 12 CPM [26].

**Figure 4:**
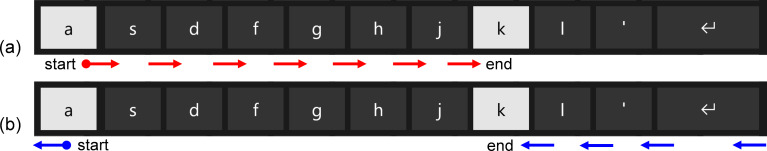
Diagram showing the two possible cursor movement directions to go from letter “A” to “K” in a standard QWERTY keyboard layout; path (a) could be implemented by seven swift left eye movements, and path (b) provides a faster route to reach the same destination by five swift right eye movements.

If the eye mouse is to be used only to facilitate the typing on a virtual keyboard, then, to readily increase the typing speed even for a non-trained user, some software-level realizations can be implemented by applying a fixed coordinate system. That is, the cursor moves only to specific locations of the buttons of the virtual keyboard. For example, in a standard QWERTY keyboard, instead of performing one swift left eye movement to initiate continuous movement of the cursor from letter “A” to letter “K,” one can perform seven swifts left movements to traverse the cursor one at a time to its nearest neighbor in the initiated direction of movement (Figure 4a). Alternatively, five swift right movements can be performed to do the same task but in a comparatively faster way (Figure 4b).

To illustrate this, five volunteers have participated in a study to test the system by writing randomly generated six-letter sequences, summarized in Table 2, together with the typing duration. To train the volunteers, they were shortly briefed about which eye movement corresponds to which action, and they were asked to explore the device before the actual experiment, by trying to write their names. All volunteers reported they felt comfortable using the device shortly within merely 2–3 min. The shortest typing duration was 55 s, and the longest was 76 s, with an average typing speed of ca. 62 s for six characters or approx. 6 CPM. By implementing letter and word prediction algorithms, a further increase in typing speed can be expected [27].

**Table 2:** Summary of generated random letter sequences with corresponding typing duration using eye movements.

Subject	Random letter sequence	Duration [s]

1	p h n e x v	55
2	g z n b a y	69
3	l a w x s t	58
4	e m j b c b	53
5	g h e b v z	76

**Figure 5:**
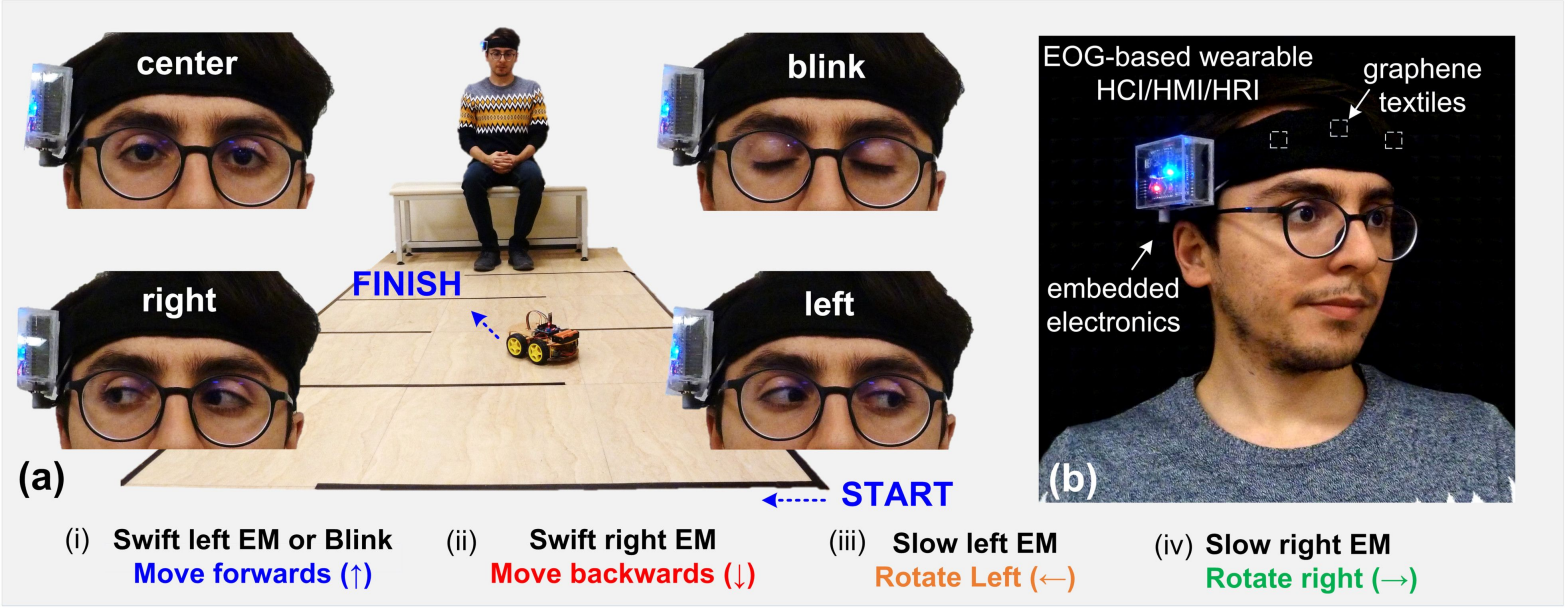
Overview of the second technology demonstrator for the wearable graphene textile-based assistive device for HMI applications. A four-wheeled robot is remotely controlled and steered in a maze. (a) Scenario-specific eye movements (EMs) allow one to steer the robot in different directions as follows: (i) swift left EM or blinking trigger forward motion (↑), (ii) swift right EM triggers backward motion (↓), (iii) slow left EM triggers rotation to the left (←), (iv) slow right EM triggers rotation to the right (→). Insets show images of the eye during different movements. This includes eyes when fixated at the center, while performing a voluntary blink, while performing a right move and fixated at the right, and while performing a left move and fixated at the left; (b) picture of the wearable eye tracker including smart headband with graphene textile electrodes and embedded electronics housed by an acrylic holder all mounted on the head.

In recent years, there have been many studies on skin-compatible, body-worn devices, collectively referred to as wearable electronics, for applications ranging from energy harvesting to human health and motion monitoring [28–34]. In a second demonstration, we have investigated the potential of the developed graphene textile-based wearable system as an assistive device to remotely control objects, including the steering of a wheelchair using eye movements, which is critical for ALS patients. To show the feasibility of the developed system, the motion of an Arduino-based, custom-designed four-wheeled robot was controlled (Figure 5). In this experiment, eye blink and swift left and right eye movements initiate forward and backward motions, while slow right and left movements enable rotation to the right and left sides.

**Figure 6:**
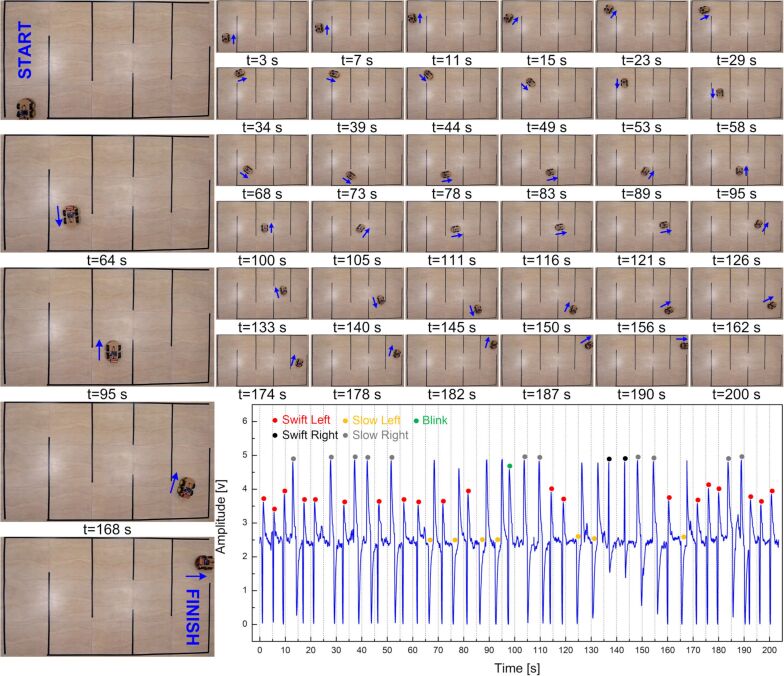
Snapshots of the robot car at different instances and plot of the recorded EOG trace during steering of the robot over a duration of more than 3 min with interpretations for the different eye movements (EMs) labeled in the waveform (video available in Supporting Information File 1).

To record the signals, both the robot and the acquisition unit were paired with a laptop running the previously designed GUI with slight modifications for enabling simultaneous communication of the GUI with robot and EOG acquisition unit. However, in actual usage cases, the laptop can be eliminated by directly connecting the EOG unit to the robot. At the robot end, incoming commands are delivered to a DC motor driver (L298, STMicroelectronics, Switzerland) in the form of pulse-width modulation (PWM) signals with a configured duty cycle value (i.e., speed), which can be changed through settings. To simplify HRI and to improve the user control experience, forward/backward movements and rotation of the robot were preset at a given distance (ca. 15 cm) and a given angle (ca. 45°), both of which can be adjusted in the GUI. Based on this configuration, eye movements were used to steer the robot along the exit path of a maze without crossing the boundaries marked with black tape (Figure 6).

A total of 41 eye movements including 19 swift left EMs (forward), two swift right EMs (backward), seven slow left EMs (turn left), eleven slow right EMs (turn right), and a single blink (forward) action were performed and recorded over a duration of ca. 200 s. Analysis of the extracted video frames along with the recorded EOG waveforms revealed a detection accuracy of approx. 98%. Only one out of 41 eye movements was misinterpreted with the developed system, demonstrating the near excellent performance of the truly wearable headband prototype for human–robot interaction and control.

## Conclusion

In contrast to well-established vision-based gaze tracking, we demonstrate electrooculography (EOG) with truly wearable graphene textiles, enabling an effective, low-cost, low-power, body-worn embedded system with small form factor to monitor and utilize eye movements for the remote control of objects. EOG allows for a recording of ocular biopotentials regardless of ambient lighting conditions, camera line of sight, or the presence of obstacles. Even when the subject’s eyes are closed, seamless detection and harnessing of eye movements for object control without the need for a camera is possible. Along with the advantages of being soft and wearable in the form of a smart textile-based accessory with peripheral electronics, the developed EOG system can be utilized in virtual reality environments and serve as a valuable communication platform for people with disabilities such as amyotrophic lateral sclerosis (ALS). In this study, we also demonstrated a proof of concept of “active” graphene textile electrodes and achieved initial results that warrant room for detailed investigation. Additionally, in the current work, the information obtained from EOG remains coarse, the users are static, and signal processing is yet to be optimized for mobile scenarios, which indicates future research directions. We envision that further developments will be possible with the results presented in this work, which lay down the foundations regarding the use of wearable graphene textiles in control applications tailored explicitly to EOG-based human–computer/robot interaction (HCI/HRI).

## Supporting Information

File 1 Video showing the steering and control of robot car.
